# Sexual Dimorphism and Morphological Modularity in *Acanthoscelides obtectus* (Say, 1831) (*Coleoptera*: *Chrysomelidae*): A Geometric Morphometric Approach

**DOI:** 10.3390/insects12040350

**Published:** 2021-04-14

**Authors:** Sanja Budečević, Uroš Savković, Mirko Đorđević, Lea Vlajnić, Biljana Stojković

**Affiliations:** 1 Institute for Biological Research “Siniša Stanković”—National Institute of the Republic of Serbia, University of Belgrade, Bulevar Despota Stefana 142, 11000 Belgrade, Serbia; savkovic.uros@ibiss.bg.ac.rs (U.S.); mirko.djordjevic@ibiss.bg.ac.rs (M.Đ.); 2 Institute of Zoology, Faculty of Biology, University of Belgrade, Studentski Trg 16, 11000 Belgrade, Serbia; lea.vlajnic@bio.bg.ac.rs (L.V.); bilja@bio.bg.ac.rs (B.S.)

**Keywords:** *Acanthoscelides obtectus*, geometric morphometrics, sexual dimorphism, modularity, size, shape

## Abstract

**Simple Summary:**

The seed beetle *Acanthoscelides obtectus* used in this study is a worldwide pest species that inhabits storage facilities and fields of beans. Knowing that sexual dimorphism is very common among insects, we investigated the level of morphological differences between the sexes. Such an approach allowed us to look into the modular organization of this organism. As expected, the females were larger than the males. The observed two modular organization (thorax and abdomen) was sex specific, indicating that reproductive function has the central role in forming the patterns of modularity. It seems that natural selection is driving force for females, while males are influenced more by sexual selection.

**Abstract:**

Sexual dimorphism and specific patterns of development contribute in a great manner to the direction and degree of the sexual differences in body size and shape in many insects. Using a landmark-based geometric morpohometrics approach, we investigated sex-specific morphological size and shape variation in the seed beetle, *Acanthoscelides obtectus*. We also tested the functional hypothesis of the two morphological modules—thorax and abdomen in both sexes. Female-biased sexual dimorphism in size was shown, while differences in shape were reflected in the wider thorax and abdomen and shorter abdomen in females in comparison to males. The functional hypothesis of a two-module body was confirmed only in females before correction for size, and in both sexes after the allometry correction. Our results indicate that reproductive function has the central role in forming the patterns of modularity. We hypothesize that high morphological integration of the abdomen in females results from intense stabilizing selection, while the more relaxed integration in males is driven by the higher intensity of sexual selection.

## 1. Introduction

Seed beetles (*Bruchidae*) are phytophagous, holometabolous insects with a worldwide distribution and often are major pests of legume plant species [1]. Within bruchine species there is a great variation in body shape and size due to genetic variability, but also as a result of adaptive responses to different ecological factors, i.e., geographical distribution [2], environmental variables such as temperature [3], or insect shift to novel host plant species [4]. In addition, one of the most remarkable sources of morphological variation refers to the direction and degree of sexual differences in body size and shape resulting in sexual dimorphism [5,6,7]. The most common pattern of sex differences among insects is female-biased size dimorphism, in which larger females have adaptive advantages such as greater fecundity, fertility and, in some cases, better parental care [8]. However, different results have been reported in seed beetles from the *Chrysomelidae* family (i.e., *Stator limbatus* (Horn, 1873)) [9]. It has been hypothesized that bigger males have large amounts of sperm nutrient which are transferred to females, raising their fecundity. Although the data on the size dimorphism between females and males are abundant and the factors that contribute to the sex size differences are well examined, studies related to coleopteran sexual shape dimorphism are underexplored [5,7,10,11].

Morphological integration refers to functional, developmental and/or evolutionary connection between organism’s morphological traits [12]. More closely, developmental processes that underlie phenotypic variation usually simultaneously encompass several morphological traits that share the same genetic basis, developmental paths or a function, and cause a certain degree of internal integration between them [13]. Such integration between a group of traits leads to the development of a morphological module, which is relatively less linked to other integrated modules [12,14]. This is the concept of modularity which focuses on relative differences in the level of the integration of the parts within and between modules of organism traits and therefore can be applied to address important evolutionary questions [15,16,17,18]. The evolutionary significance of a modular biological organization lays in a potentially enhanced evolvability, that is, the increased ability of organisms to evolve and respond to different selective challenges [19]. In other words, selection is able to act on each of these distinctive entities separately without great interference [20].

The concept of modularity in seed beetles’ body plans could be illustrated by three easily identified separate entities integrated through their function, with a degree of independence between each other: head, thorax and abdomen. The size and shape of the head are adapted to feeding habits, while the thorax is specialized for locomotion [21]. The thorax encompasses muscles for flight and for the movement of legs and body segments, and thus has a major role in locomotion [22]. Finally, the abdomen of beetles is linked to the reproduction, since it contains the reproductive organs and all the nutrients and energy reserves that can be used for producing eggs and ejaculate [23]. Additionally, taking into account that the size and shape of abdomen could be an important trait for mate choice in beetles, abdominal morphological variation is expected to be under sexual selection [24].

In this research we analyzed sexual dimorphism and morphological modularity in one holometabolous, bruchine species—*Acanthoscelides obtectus* (Say, 1831). Classic morphological analyses pointed out that sexual dimorphism in *A. obtectus* is related to the last segment of the abdomen (fifth sternite) and the orientation of the pygidium [25,26]. However, these studies have been limited to a few measures. A landmark-based geometric morphometric is a far more powerful tool that allows quantifying and visualizing shape variation, providing precise information on interindividual and intraindividual morphological variability [18,27]. In addition, this approach enables analyzing allometry, which is defined as a relationship between changes in body shape and changes in body size [28]. Exploring allometry shape changes is of great importance in studying sexual shape dimorphism and the detection of modularity, because allometry can have major effects on the patterns of variation and integration [11,18,29]. By applying a geometric morphometric approach we explored and tested: (i) the specific morphological differences in size and shape between *A. obtectus* females and males; (ii) the influence of size on body shape changes; and (iii) the functional hypothesis of the two modules: thorax and abdomen in females and males separately.

## 2. Materials and Methods

### 2.1. Study Species—Seed Beetle (Acanthoscelides obtectus), Laboratory Population and Rearing Conditions

This research was conducted on seed beetles (*A. obtectus*) from a laboratory population (hereafter referred to as base) maintained for more than 35 years (301 generations) under constant conditions (30 °C ± 0.1 °C, relative humidity 30% ± 1%). Base population was established using beetles that hatched from infected bean seeds from three legume storages. In order to limit the severe effects of inbreeding, at least 600 randomly sampled individuals contributed to the consecutive generation. Individuals from different generations were not mixed, i.e., there was no generation overlap.

According to the laboratory protocol, all insects were reared in the dark incubator set at 30 ± 1 °C. Since *A. obtectus* is facultative aphagous, food and water was not offered to adults. Food for larvae was pesticide free, organic white bean seeds that were frozen for 48 h on −20 °C before use in order to evade potential contamination.

### 2.2. Collection of Samples

Immediately upon emergence, a total of 314 adults from base population (157 females and 157 males) were collected and stored on −20 °C in a single day. Adults were set up on plasticine mold glued to a microscope plate and photographed with Nikon Digital Sight Fi2 Camera attached to Nikon SMZ800 against a scale bar 10 mm on ventral side. The distance and magnification were kept constant during photographing.

### 2.3. Landmark Data

**Figure 1:**
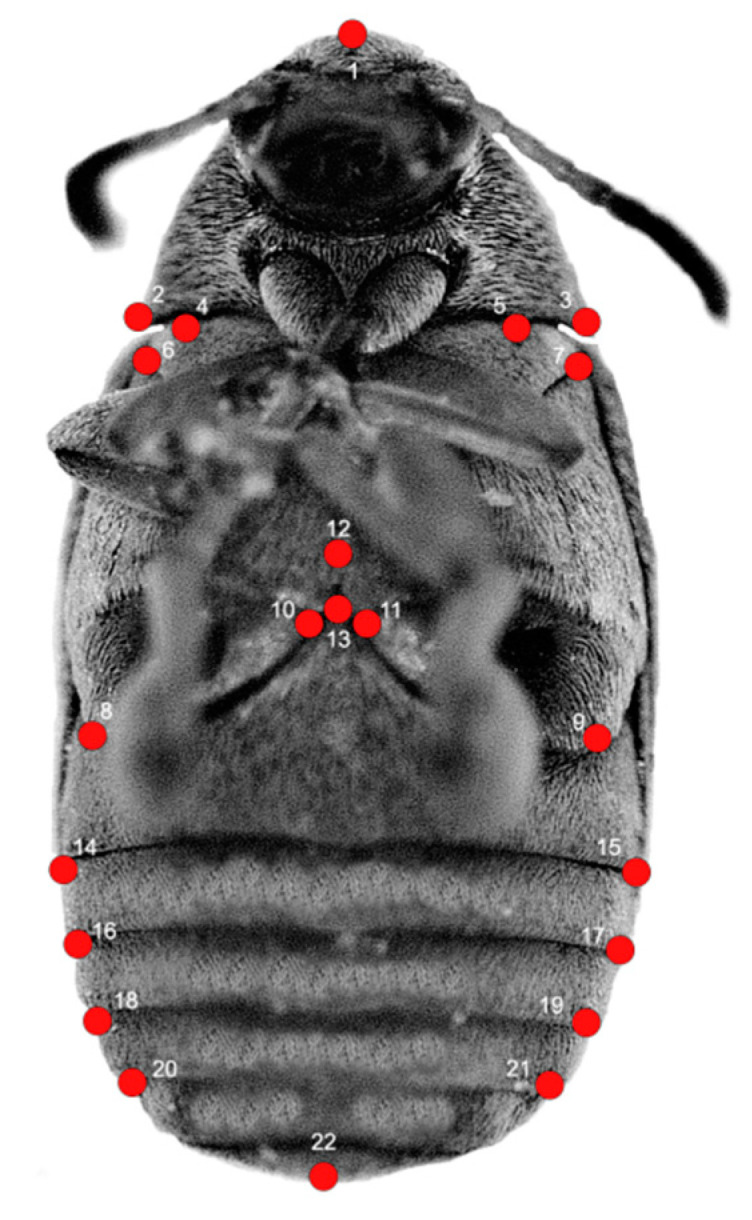
Landmark configurations on *Acanthoscelides obtectus* example. See Table 1 for landmark definitions.

To characterize the shape of body, we applied the methods of geometric morphometrics, which use the relative positions of the set of landmarks to quantify morphological variation [19,30,31]. We selected configurations of 22 landmarks of objects (12 landmarks for the thorax and 10 landmarks for the abdomen) (Figure 1, Table 1). Potential differences in the shape of head and specialized mouth parts (e.g., mandibles) go beyond the scope of this study. Therefore, this part of the body has not been covered with landmarks and included in analyses. The landmarks were digitized by one person in TpsDig2 software [32].

### 2.4. Geometric Morphometric Analyses

#### 2.4.1. Analyses of Size and Shape Variation Patterns

Centroid size, the square root of the sum of squared distances of all the landmarks from their centroid, was used as a measure of size of the seed beetle’s body [31]. The differences in the body sizes between females and males were tested by one-way ANOVA. Statistical analyses of centroid size were carried out using GLM procedure of SAS statistical software [33].

To extract shape variables from the landmark configuration of beetle’s body, we used Procrustes superimposition to eliminate effects of size, position and orientation [31]. We applied principal component analysis (PCA) on the covariance matrices of shape variables to describe overall shape variation pattern of the seed beetle’s body in females and males [27].

In order to explore and visualize allometric shape changes, we performed multivariate regression analyses of obtained shape variables for females and males [34]. Statistical significance of allometric shape changes were obtained by permutation test (10,000 iterations). Residuals obtained from these regression analyses represent nonallometric component of shape variation.

To quantify differences in shape between females and males we calculated Procrustes distances (PD)—the square root of the squared distances between pairs of corresponding landmarks. This procedure was repeated on nonallometric component of shape variation. The statistical significance of PD was obtained using the permutation test (10,000 iterations).

To estimate the measurement error, we used Procrustes ANOVA with the main effects of: (i) individuals—indicating the interindividual phenotypic differences in shape of females and males; and (ii) residual term representing the measurement error [35]. For this analysis, the whole sample was digitized twice by one person.

#### 2.4.2. Analyses of Shape Covariation Patterns

To test the two module hypothesis of modularity of beetles’ females and males, we used the covariance matrices of pairways Procrustes distances. As a measure of strength of association between the hypothesized partitions, RV coefficients (ratio that describes the degree of covariation between sets of variables relative to the variation and covariation within sets of variables) were calculated and compared with RV coefficients obtained for all possible alternative partitions [29,36]. Values of the RV coefficient can range from 0 to 1, providing that lower values indicate weaker correlation [36]. If a value of the RV coefficient between hypothesized modules is smaller than for most of all alternative partitions, the modularity hypothesis will be confirmed [29]. RV coefficients for females and males were calculated twice: for allometric and nonallometric component of the shape variation. All statistical analyses and visualizations of shape changes were conducted using MorphoJ software package [37].

## 3. Results

### 3.1. Sexual Dimorphism in Size and Shape

**Figure 2:**
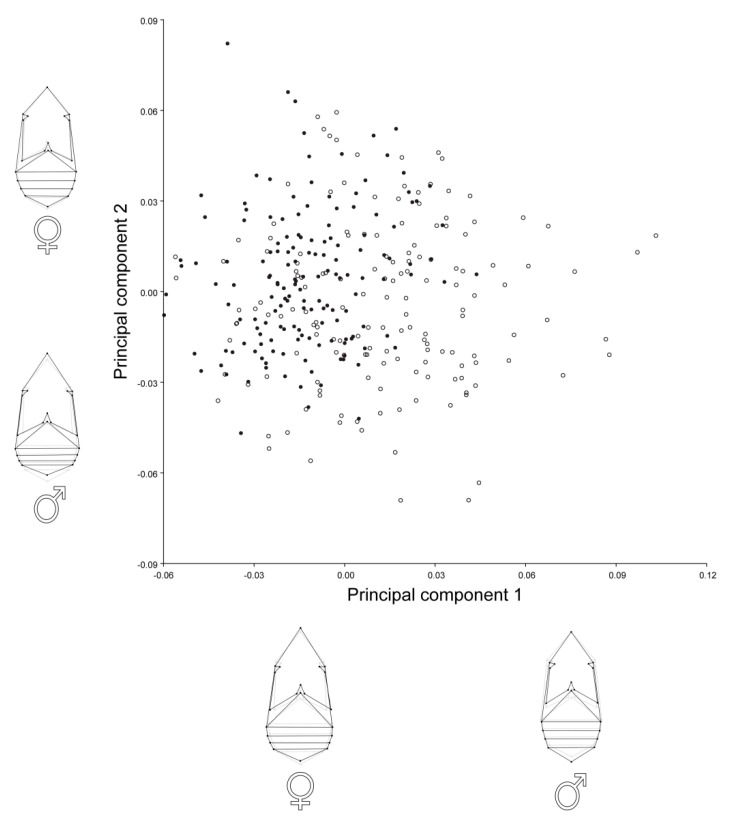
Principal component analysis (PCA) of body shape of *Acanthoscelides obtectus* females and males. Scatterplot of the PCs 1 and 2 accounted together for about 55% of the overall shape variation. Filled circles are assigned for females, while open circles are assigned for males. Shape changes of females and males associated with the PCs are represented by wireframe graphs.

The principal component analysis (PCA) for females and males showed that the first two principal components (PC1 and PC2) described about 55% of the total shape variation (Table 2). The main patterns of the shape variation were changes in the relative length vs. width of the thorax and abdomen (Figure 2). PC1 and PC2 in females were associated with shortening of the abdomen and slight elongation of the thorax. In males, PC1 reflected elongation of abdomen and narrowing and shortening of the thorax, while PC2 was related to a shortening of abdomen and elongation and widening of the thorax. A scatterplot of PC scores revealed clear tendency of differences in body shape between A. obtectus females and males (Figure 2).

Multivariate regression of shape variables on size showed highly statistical significance and explained different portion of the total variation for females (4.18%, *p* < 0.0001) and males (13.88%, *p* < 0.0001).

The one-way ANOVA indicated significant sex-specific differences of seed beetles (*F* = 109.59; d.f. = 1; *p* < 0.0001). Mean centroid sizes indicated that females (mean CS_females_ = 7.711) are larger than males (CS_males_ = 7.641).

**Figure 3:**
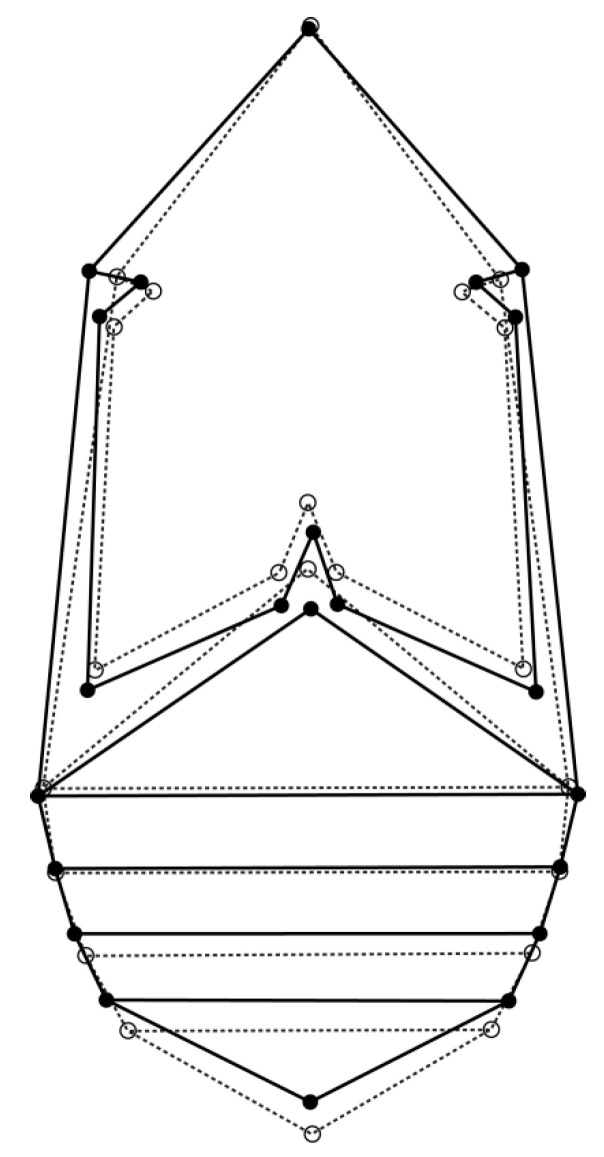
Discriminant functional analyses of seed beetles. The outline graphs show differences in shape between *Acanthoscelides obtectus* females (solid line) and males (dashed line).

For the allometric and nonallometric component of shape variation, the permutation test revealed high statistical significance (*p* < 0.0001) for Procrustes distances between females and males (PD_allometric_ = 0.028, PD_non-allometric_ = 0.014). Visualization of the sexual shape dimorphism is presented by a diagram in Figure 3. The differences in shape between the sexes are characterized by a wider thorax in general, and an elongated metathorax in females in comparison to males. Females’ first abdominal sternite is shortened, while females’ 2nd abdominal sternite is wider than males’. The last abdominal sternite is shorter in females.

### 3.2. Modularity

Procrustes ANOVA revealed that mean squares of interindividual variation have significant higher values than measurement error (Table 3), so Procrustes distances can be used as valid variables for testing hypothesis of modularity.

**Figure 4:**
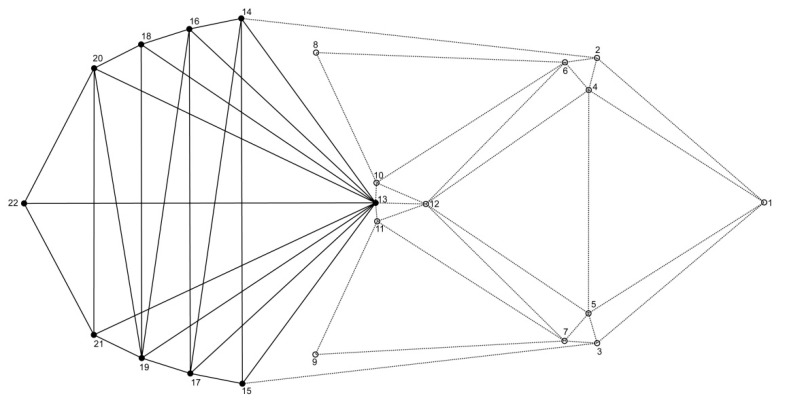
A priori hypothesis of a two-module (thorax—dashed line, abdomensol—id line) organization of female and male seed beetles.

**Figure 5:**
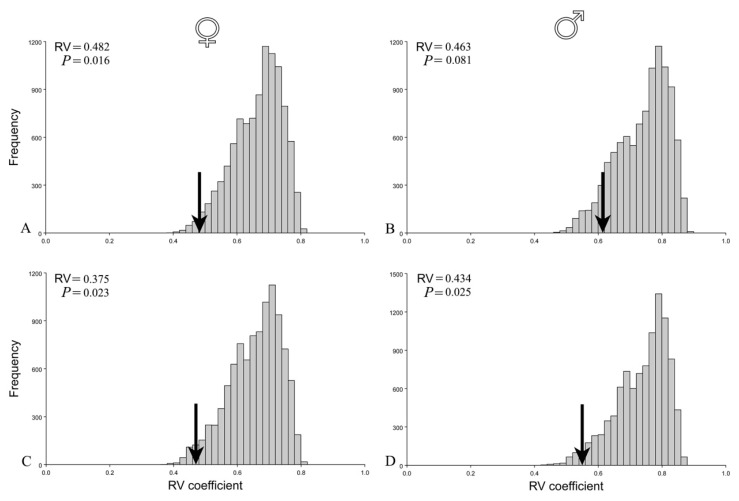
Evaluation of a two-module hypothesis of female and male seed beetles before (**A**,**B**) and after correction for size (**C**,**D**). Arrows on histograms of distribution of the RV coefficients for the alternative partitions indicate values of RV coefficients for the hypothesis. Statistical significance of tested hypothesis is presented by *p* values.

The functional hypothesis of the two modules (thorax and abdomen) (Figure 4) was confirmed for females, but not for males before the correction for size (Figure 5A,B). Covariation between thorax and abdomen in females was among the lowest when compared with covariation for alternative partitions (*p* = 0.016). On the other hand, in males, the hypothesis of the two modules was not confirmed (*p* = 0.081). Interestingly, after removing the influence of allometry, the correlation matrices of the residuals were statistically significant and correlated with theoretically derived matrices for both females (*p* = 0.023) and males (*p* = 0.025) (Figure 5C,D). Hence, after correction for size, the two module functional hypothesis was confirmed for both sexes.

## 4. Discussion

### 4.1. Morphological Variation and Sexual Dimorphism in Size and Shape

In the present work, we quantified and compared female and male morphological variation in *A. obtectus*. Our results revealed the female-biased size dimorphism that is a common pattern in insects. It is expected for sexual dimorphism in insects to be affected by both life-history evolution and sexual selection. Larger female body size is usually related to the fitness, that is, to the higher number of viable offspring and an increase in mating success [38,39,40]. In many insect species, as well as in *A. obtectus* [41,42], it has been confirmed that large females are able to convert higher portions of their accumulated resources into fecundity [43,44,45]. Evolutionary changes of body size in insects could also be achieved via selection on development time, being that the time needed for the development of an adult is positively correlated with its body size [42,46]. Namely, in protandrous insects, the slower development of female sex leads to larger adults and their higher fecundity, whereas faster male development, although it results in smaller individuals, increases the chances for males to copulate with newly hatched females and therefore could increase male reproductive success [42].

The evolution of the female-bias size dimorphism in *A. obtectus* could also be related to the behavior during copulation. It has been shown that in this species courtship activities are simple; they do not include specific rituals or acoustic signals, and the most important activity for the males is the level of their aggressiveness and persistence in chasing females [47]. In general, copulation in insects often has harmful effects in females, resulting in reduced fitness and even death [48]. If smaller males could potentially do less harm to females during copulation, then sexual selection could influence the general size and shape of males as well as to select for females that are better in recognizing the least harmful males [49].

Sexual dimorphism in the shape of abdomen has been confirmed in different groups of insects, such as *Scathophagidae* [49], and, recently, the *Carabidae* family [5,7]. The most pronounced sex difference in shape that we observed in *A. obtectus* was divergence in the abdomen, which was wider and shorter in females than in males. Such specific shape can be related to the ability of females to accumulate and transport more eggs [39,50]. Hence, the shape of the female’s abdomen and fecundity can be positively correlated through natural selection [24,51]. Again, the specific shape of the abdomen in this species could be the result of specific patterns of sexual selection, especially related to its reproductive behavior. Unlike many insect species, contact between females and males during copulation is loosed in *A. obtectus* enabling females to avoid injuries relatively easily [52]. The particular size and shape of female and male abdomens allow the copulation to be efficient enough for potentially a short period of time. Accordingly, the more elongated body of *A. obtectus* males can assist in forced copulation with females [53] and more accurate positioning above females just before copulation [52].

### 4.2. Modularity

Research on morphological shape variation in holometabolous adults, integration and modularity are mostly limited to *Carabidae* and *Hymenoptera* [5,54,55]. The origin of morphological integration and modularity has been analyzed in different insect species on various body parts: mouthparts in *Pterostichus thunbergi* (Morawitz, 1862) [56], hind wings in *Diabrotica virgifera virgifera* (LeConte, 1868) [57], wings in bumblebees [58], dragonflies [59] and in adult ants [54]. In our study, the hypothesis of the two functional modules (thorax and abdomen) was confirmed for *A. obtectus* females before correction for size, and for both sexes after size correction.

One common explanation for the evolution of tight integration of traits within a module refers to a strong stabilizing selection acting on the functionality of the module [60]. Although the existence of distinctive entities enables their evolution independently from other body parts, this also constrains fast diversification of a module in order to maintain conserved modes of function. Genetically and/or environmentally induced perturbations during development, which could alter the basic functional structure, have to be limited in their effects on a module. It seems that the female abdomen in *A. obtectus* is under strong selective pressure due to its importance in reproductive function and it is independent from body size [61]. On the other hand, the shape of male abdomen is significantly associated with body size, indicating lower integration of male modules. Thus, we hypothesize that male abdominal parts could have greater potential for the evolution of diverse shapes and structures because they are more driven by sexual selection. Being that the reproductive successes of males is highly dependent on their ability to mount females, it could be suggested that different abdominal sizes need different abdominal shapes in order to achieve copulation.

Our results on *A. obtectus* lead to conclusion that evolution of modules of body parts with reproductive function are under the influence of both natural and sexual selection, but the courses and intensity of these mechanisms are different in females and males. In support of that is the recent morphological study on green-belly stink bug (*Dichelops melacanthus* (Dallas, 1851) (*Heteroptra*: *Pentatomidae*)) genitalia which strongly indicates that the reproductive organs are subjected to sex specific selection, although male and female genitalia are functionally associated [62]. The authors hypothesized that integration of reproductive organs in females constrains diversification via stabilizing selection, while in males directional selection is more responsible for their maintenance or for the improvement of copulatory performance. It seems that sex specific modular patterns may be of greater importance in the evolution of different insect species than previously thought.

## 5. Conclusions

- Female-biased size dimorphism in seed beetle (*Acanthoscelides obtectus*) laboratory population is affected by both life-history and sexual selection.
- Females have shorter and wider abdomens compared to more elongated abdomens in males.
- By testing the modularity hypothesis it was confirmed that female and male body is compartmentalized into two functional modules: thorax and abdomen. The integration of the abdomen in males is dependent on their size, indicating the more prominent role of sexual selection. On the other hand, strong modularity in females, regardless of size, is the result of strong natural selection on reproductive function.

## Figures and Tables

**Table 1:** Definitions of landmarks on the *Acanthoscelides obtectus*.

Landmark Number	Landmark Position
1.	Point on the top of pronotum
2.	Leftmost point on pronotum
3.	Rightmost point on pronotum
4.	Highest point on mesosternum on left side
5.	Highest point on mesosternum on right side
6.	Point on top of metasternum on left side
7.	Point on top of metasternum on right side
8.	Point on bottom of metacoxa on left side
9.	Point on bottom of metacoxa on right side
10.	Point on metacoxa and metasternum joining on left side
11.	Point on metacoxa and metasternum joining on right side
12.	Center of metathorax
13.	Point on the top of 1st sternite
14.	Leftmost point on 1st and 2nd sternite joining
15.	Rightmost point on 1st and 2nd sternite joining
16.	Leftmost point on 2nd and 3rd sternite joining
17.	Rightmost point on 2nd and 3rd sternite joining
18.	Leftmost point on 3rd and 4th sternite joining
19.	Rightmost point on 3rd and 4th sternite joining
20.	Leftmost point on 4th and 5th sternite joining
21.	Rightmost point on 4th and 5th sternite joining
22.	Point on half on 5th sternite bottom margin

**Table 2:** Eigenvalues and contribution of principal components (PCs) in the shape variation of *Acanthoscelides obtectus* females and males.

	Eigenvalues	% Variance	Cumulative %
**Females**			
PC l	0.00057	27.185	27.185
PC 2	0.00037	17.603	44.787
Total variance	0.00208		
**Males**			
PC l	0.00089	31.681	31.681
PC 2	0.00073	25.533	57.214
Total variance	0.00283		

**Table 3:** Procrustes ANOVA of shape in *Acanthoscelides obtectus* females and males with effects of individual (interindividual variation) and measurement error. SS—sum of squares, MS—mean of the sum of squares, df—degree of freedom, F—value of F test, *p*—level of statistical significance.

	SS	MS	df	F	*p*
**Females**					
Individual	0.46976	0.0001506	3120	3.31	<0.0001
Error	0.03758	0.0000060	6280		
**Males**					
Individual	0.70243	0.0002251	3120	4.59	<0.0001
Error	0.02821	0.0000045	6280		
